# Piperidinium 3-carb­oxy-4-hydroxy­benzene­sulfonate monohydrate

**DOI:** 10.1107/S1600536808014256

**Published:** 2008-05-17

**Authors:** Zhenhuan Li, Bowen Cheng, Su Kunmei

**Affiliations:** a College of Materials and Chemical Engineering, and Tianjin Key Laboratory of Fiber Modification & Functional Fiber, Tianjin Polytechnic University, Tianjin 300160, People’s Republic of China

## Abstract

The asymmetric unit of the title compound, C_5_H_12_N^+^·C_7_H_5_O_6_S^−^·H_2_O, contains a piperidinium cation, one 3-carb­oxy-4-hydroxy­benzene­sulfonate anion and one water mol­ecule. Inter­molecular O—H⋯O, O—H⋯S and N—H⋯O hydrogen bonds generate a three-dimensional hydrogen-bonded framework.

## Related literature

For related literature, see: Smith *et al.* (2007 ▶).
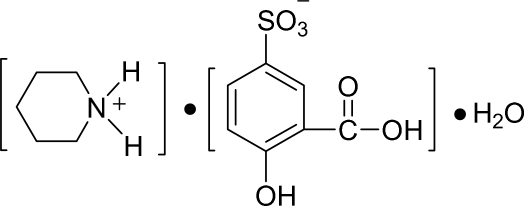


## Experimental

### 

#### Crystal data

C_5_H_12_N^+^·C_7_H_5_O_6_S^−^·H_2_O
*M* *_r_* = 321.34
Monoclinic, 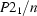

*a* = 6.8895 (14) Å
*b* = 13.202 (3) Å
*c* = 16.255 (3) Å
β = 93.739 (3)°
*V* = 1475.3 (5) Å^3^
*Z* = 4
Mo *K*α radiation
μ = 0.25 mm^−1^
*T* = 294 (2) K
0.24 × 0.20 × 0.16 mm

#### Data collection

Bruker SMART CCD area-detector diffractometer
Absorption correction: multi-scan (*SADABS*; Sheldrick, 1996 ▶) *T*_min_ = 0.942, *T*_max_ = 0.961
7480 measured reflections
2602 independent reflections
1966 reflections with *I* > 2σ(*I*)
*R*_int_ = 0.031

#### Refinement

*R*[*F* ^2^ > 2σ(*F* ^2^)] = 0.035
*wR*(*F* ^2^) = 0.091
*S* = 1.04
2602 reflections
209 parameters
3 restraints
H atoms treated by a mixture of independent and constrained refinement
Δρ_max_ = 0.26 e Å^−3^
Δρ_min_ = −0.28 e Å^−3^

### 

Data collection: *SMART* (Bruker, 2000 ▶); cell refinement: *SAINT* (Bruker, 2000 ▶); data reduction: *SAINT*; program(s) used to solve structure: *SHELXS97* (Sheldrick, 2008 ▶); program(s) used to refine structure: *SHELXL97* (Sheldrick, 2008 ▶); molecular graphics: *SHELXTL* (Sheldrick, 2008 ▶); software used to prepare material for publication: *SHELXTL*.

## Supplementary Material

Crystal structure: contains datablocks I, global. DOI: 10.1107/S1600536808014256/gw2042sup1.cif

Structure factors: contains datablocks I. DOI: 10.1107/S1600536808014256/gw2042Isup2.hkl

Additional supplementary materials:  crystallographic information; 3D view; checkCIF report

## Figures and Tables

**Table 1:** Hydrogen-bond geometry (Å, °)

*D*—H⋯*A*	*D*—H	H⋯*A*	*D*⋯*A*	*D*—H⋯*A*
O7—H7*B*⋯O1	0.855 (9)	1.933 (10)	2.786 (2)	176 (2)
O7—H7*A*⋯O2^i^	0.859 (9)	1.912 (10)	2.770 (2)	177 (2)
N1—H1*B*⋯O5	0.88 (3)	2.55 (2)	2.983 (3)	110.6 (18)
N1—H1*B*⋯O7^ii^	0.88 (3)	2.16 (3)	2.996 (3)	157 (2)
N1—H1*A*⋯O2^iii^	0.92 (3)	1.90 (3)	2.807 (3)	170 (2)
O6—H6⋯O5	0.84 (3)	1.82 (3)	2.597 (2)	153 (3)
O4—H4⋯O7^ii^	0.85 (3)	1.75 (3)	2.601 (2)	179 (3)

Symmetry codes: (i) 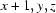; (ii) 
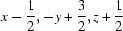
; (iii) 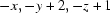.

**Fig. 1.**
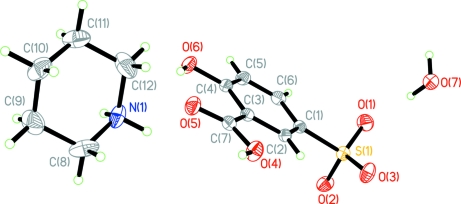
The asymmteric unit of title compound. Displacement ellipsoids are drawn at the 30% probability level.

**Fig. 2.**
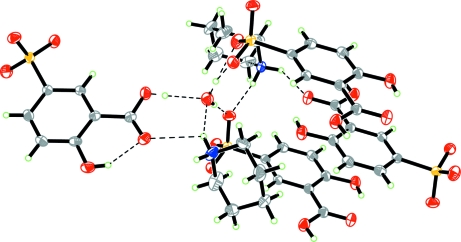
Part of the crystal packing of the title compound. O—H···O and N—H···O hydrogen bonds are shown as dashed lines.

## supplementary crystallographic information

### Comment

5-Sulfosalicylic acid (SSA) has six potential donor sites in the three substituent groups (the sulfonic acid, the carboxylic acid and the phenolic groups), and it gives mono-, di- and trianionic ligand species through deprotonation. The presence of numerous oxygen atoms in the substituent groups usually results in hydrogen-bonding associations, and the self-assembly process of crystallization often requires the incorporation of water molecules in the structures (Smith *et al.* 2007). We report here the crystal structure of the title compound.

The asymmetric unit of the title compound contains one piperidium cation cation, one 3-carboxyl-4-hydroxyl-benzenesulfonate anion and one water molecule (Fig. 1). The bond distances and angles in the cationic and anionic species are normal. An intramolecular O6—H6···O5 hydrogen bond is observed. The molecular packing (Fig. 2) is stabilized by intermolecular O—H···O, O—H···S and N—H···O hydrogen bonds (Table 1), These interactions generate a three-dimensional hydrogen-bonded framework structure.

### Experimental

2-Hydroxy-5-sulfobenzoic acid (2.18 g, 10 mmol), piperidine (0.85 g, 10 mmol) and H_2_O (20 ml) were loaded into a 50 ml roundbottom flask, and heated to dissolve the solid. Crystals of the title compound were obtained by slow evaporation of deionic H_2_O solution.

### Refinement

The H atoms of the water molecule, and the N-bound H atom H atom were located in a difference Fourier map, and refined with the O—H and N—H distance restraints of 0.86 (1) and 0.90 (1) Å, respectively. All other H atoms were positioned geometrically [O—H = 0.82 Å (hydroxyl), C—H = 0.93 Å (aromatic) and 0.96 Å (methyl)] and refined using a riding model, with *U*_iso_(H) = 1.5*U*_eq_(carrier) for hydroxyl and methyl H atoms and 1.2*U*_eq_(C) for other H atoms.

### Crystal data

**Table:**

C_5_H_12_N^+^·C_7_H_5_O_6_S^–^·H_2_O	*F*_000_ = 680
*M**_r_* = 321.34	*D*_x_ = 1.447 Mg m^−^^3^
Monoclinic, *P*2_1_/*n*	Mo *K*α radiation λ = 0.71073 Å
Hall symbol: -P 2yn	Cell parameters from 2608 reflections
*a* = 6.8895 (14) Å	θ = 3.0–25.1º
*b* = 13.202 (3) Å	µ = 0.25 mm^−^^1^
*c* = 16.255 (3) Å	*T* = 294 (2) K
β = 93.739 (3)º	Stick, colourless
*V* = 1475.3 (5) Å^3^	0.24 × 0.20 × 0.16 mm
*Z* = 4	

### Data collection

**Table:**

Bruker SMART CCD area-detector diffractometer	2602 independent reflections
Radiation source: fine-focus sealed tube	1966 reflections with *I* > 2σ(*I*)
Monochromator: graphite	*R*_int_ = 0.031
*T* = 294(2) K	θ_max_ = 25.0º
φ and ω scans	θ_min_ = 2.0º
Absorption correction: multi-scan(SADABS; Sheldrick, 1996)	*h* = −8→8
*T*_min_ = 0.942, *T*_max_ = 0.961	*k* = −15→15
7480 measured reflections	*l* = −19→10

### Refinement

**Table:**

Refinement on *F*^2^	Secondary atom site location: difference Fourier map
Least-squares matrix: full	Hydrogen site location: inferred from neighbouring sites
*R*[*F*^2^ > 2σ(*F*^2^)] = 0.035	H atoms treated by a mixture of independent and constrained refinement
*wR*(*F*^2^) = 0.091	*w* = 1/[σ^2^(*F*_o_^2^) + (0.0392*P*)^2^ + 0.5098*P*] where *P* = (*F*_o_^2^ + 2*F*_c_^2^)/3
*S* = 1.04	(Δ/σ)_max_ = 0.001
2602 reflections	Δρ_max_ = 0.26 e Å^−^^3^
209 parameters	Δρ_min_ = −0.28 e Å^−^^3^
3 restraints	Extinction correction: SHELXL, Fc^*^=kFc[1+0.001xFc^2^λ^3^/sin(2θ)]^-1/4^
Primary atom site location: structure-invariant direct methods	Extinction coefficient: 0.0191 (15)

### Special details

**Table:**

Geometry. All e.s.d.'s (except the e.s.d. in the dihedral angle between two l.s. planes) are estimated using the full covariance matrix. The cell e.s.d.'s are taken into account individually in the estimation of e.s.d.'s in distances, angles and torsion angles; correlations between e.s.d.'s in cell parameters are only used when they are defined by crystal symmetry. An approximate (isotropic) treatment of cell e.s.d.'s is used for estimating e.s.d.'s involving l.s. planes.
Refinement. Refinement of *F*^2^ against ALL reflections. The weighted *R*-factor *wR* and goodness of fit *S* are based on *F*^2^, conventional *R*-factors *R* are based on *F*, with *F* set to zero for negative *F*^2^. The threshold expression of *F*^2^ > σ(*F*^2^) is used only for calculating *R*-factors(gt) *etc*. and is not relevant to the choice of reflections for refinement. *R*-factors based on *F*^2^ are statistically about twice as large as those based on *F*, and *R*- factors based on ALL data will be even larger.

### Fractional atomic coordinates and isotropic or equivalent isotropic displacement parameters (Å^2^)

**Table:**

	*x*	*y*	*z*	*U*_iso_*/*U*_eq_	
S1	0.16322 (7)	0.89594 (4)	0.24082 (3)	0.03289 (18)	
O1	0.3120 (2)	0.93486 (13)	0.19039 (9)	0.0516 (4)	
O2	−0.0297 (2)	0.93311 (11)	0.21241 (10)	0.0483 (4)	
O3	0.1658 (2)	0.78755 (11)	0.25065 (9)	0.0479 (4)	
O4	0.1660 (2)	0.78250 (12)	0.55005 (9)	0.0464 (4)	
H4	0.160 (4)	0.752 (2)	0.5956 (17)	0.070*	
O5	0.2197 (2)	0.91602 (11)	0.63192 (9)	0.0462 (4)	
O6	0.2977 (2)	1.08950 (12)	0.56636 (10)	0.0487 (4)	
H6	0.279 (4)	1.044 (2)	0.6018 (17)	0.073*	
N1	0.1423 (3)	0.86332 (17)	0.80518 (12)	0.0481 (5)	
H1A	0.100 (3)	0.928 (2)	0.7936 (15)	0.058*	
H1B	0.124 (4)	0.8238 (18)	0.7615 (16)	0.058*	
C1	0.2116 (3)	0.95050 (14)	0.33929 (12)	0.0294 (4)	
C2	0.1907 (3)	0.89450 (15)	0.40946 (12)	0.0296 (4)	
H2	0.1565	0.8265	0.4050	0.036*	
C3	0.2204 (3)	0.93869 (15)	0.48727 (12)	0.0310 (5)	
C4	0.2711 (3)	1.04151 (15)	0.49318 (13)	0.0349 (5)	
C5	0.2947 (3)	1.09718 (16)	0.42200 (14)	0.0387 (5)	
H5	0.3302	1.1651	0.4258	0.046*	
C6	0.2656 (3)	1.05223 (15)	0.34626 (13)	0.0365 (5)	
H6A	0.2819	1.0899	0.2989	0.044*	
C7	0.2020 (3)	0.87879 (16)	0.56285 (13)	0.0345 (5)	
C8	0.0182 (4)	0.81764 (19)	0.86596 (17)	0.0630 (8)	
H8A	0.0510	0.7466	0.8732	0.076*	
H8B	−0.1172	0.8221	0.8458	0.076*	
C9	0.0467 (4)	0.8713 (2)	0.94665 (16)	0.0691 (8)	
H9A	−0.0299	0.8381	0.9868	0.083*	
H9B	0.0008	0.9405	0.9403	0.083*	
C10	0.2573 (4)	0.8719 (2)	0.97778 (15)	0.0629 (7)	
H10A	0.2722	0.9105	1.0286	0.075*	
H10B	0.3002	0.8031	0.9895	0.075*	
C11	0.3795 (4)	0.9178 (2)	0.91490 (17)	0.0648 (8)	
H11A	0.3461	0.9888	0.9081	0.078*	
H11B	0.5154	0.9136	0.9343	0.078*	
C12	0.3510 (4)	0.8657 (3)	0.83399 (17)	0.0726 (9)	
H12A	0.4242	0.9008	0.7937	0.087*	
H12B	0.4002	0.7970	0.8390	0.087*	
O7	0.6418 (2)	0.81286 (11)	0.18899 (10)	0.0456 (4)	
H7A	0.7460 (19)	0.8483 (15)	0.1972 (16)	0.068*	
H7B	0.5375 (18)	0.8480 (15)	0.1880 (17)	0.068*	

### Atomic displacement parameters (Å^2^)

**Table:**

	*U*^11^	*U*^22^	*U*^33^	*U*^12^	*U*^13^	*U*^23^
S1	0.0357 (3)	0.0317 (3)	0.0308 (3)	0.0030 (2)	−0.0010 (2)	0.0020 (2)
O1	0.0537 (10)	0.0640 (11)	0.0382 (9)	−0.0077 (8)	0.0121 (7)	0.0017 (8)
O2	0.0434 (9)	0.0428 (9)	0.0561 (10)	0.0062 (7)	−0.0162 (7)	−0.0029 (7)
O3	0.0720 (11)	0.0301 (8)	0.0405 (9)	0.0082 (7)	−0.0032 (8)	−0.0010 (7)
O4	0.0677 (11)	0.0385 (9)	0.0334 (9)	−0.0039 (8)	0.0054 (8)	0.0020 (7)
O5	0.0549 (10)	0.0521 (10)	0.0317 (9)	−0.0034 (8)	0.0037 (7)	−0.0078 (7)
O6	0.0595 (10)	0.0418 (10)	0.0445 (10)	−0.0072 (8)	0.0004 (8)	−0.0151 (7)
N1	0.0664 (14)	0.0453 (12)	0.0314 (10)	0.0145 (10)	−0.0064 (9)	−0.0087 (9)
C1	0.0253 (10)	0.0302 (11)	0.0327 (11)	0.0025 (8)	0.0022 (8)	−0.0003 (8)
C2	0.0268 (10)	0.0274 (10)	0.0347 (11)	0.0009 (8)	0.0017 (8)	−0.0035 (9)
C3	0.0243 (10)	0.0347 (11)	0.0340 (11)	0.0017 (8)	0.0014 (8)	−0.0026 (9)
C4	0.0263 (10)	0.0359 (12)	0.0422 (13)	0.0010 (9)	−0.0001 (9)	−0.0102 (10)
C5	0.0363 (12)	0.0291 (11)	0.0507 (14)	−0.0026 (9)	0.0032 (10)	−0.0027 (10)
C6	0.0332 (11)	0.0333 (12)	0.0430 (13)	0.0007 (9)	0.0034 (9)	0.0043 (10)
C7	0.0276 (11)	0.0398 (13)	0.0361 (12)	0.0019 (9)	0.0030 (9)	−0.0046 (10)
C8	0.0621 (17)	0.0474 (15)	0.0756 (19)	−0.0215 (13)	−0.0251 (14)	0.0167 (13)
C9	0.0687 (19)	0.092 (2)	0.0485 (16)	−0.0086 (16)	0.0178 (14)	0.0167 (15)
C10	0.082 (2)	0.0681 (18)	0.0357 (14)	−0.0133 (15)	−0.0139 (13)	0.0039 (12)
C11	0.0533 (16)	0.0748 (19)	0.0635 (18)	−0.0191 (14)	−0.0169 (13)	0.0147 (14)
C12	0.0524 (17)	0.107 (2)	0.0592 (18)	0.0259 (16)	0.0114 (13)	0.0104 (16)
O7	0.0447 (9)	0.0437 (9)	0.0486 (10)	−0.0015 (7)	0.0040 (8)	−0.0046 (7)

### Geometric parameters (Å, °)

**Table:**

S1—O3	1.4398 (15)	C5—C6	1.370 (3)
S1—O1	1.4478 (16)	C5—H5	0.9300
S1—O2	1.4628 (15)	C6—H6A	0.9300
S1—C1	1.767 (2)	C8—C9	1.492 (4)
O4—C7	1.309 (3)	C8—H8A	0.9700
O4—H4	0.85 (3)	C8—H8B	0.9700
O5—C7	1.224 (2)	C9—C10	1.505 (4)
O6—C4	1.350 (2)	C9—H9A	0.9700
O6—H6	0.84 (3)	C9—H9B	0.9700
N1—C8	1.477 (3)	C10—C11	1.494 (4)
N1—C12	1.483 (3)	C10—H10A	0.9700
N1—H1A	0.92 (3)	C10—H10B	0.9700
N1—H1B	0.88 (3)	C11—C12	1.485 (4)
C1—C2	1.375 (3)	C11—H11A	0.9700
C1—C6	1.396 (3)	C11—H11B	0.9700
C2—C3	1.396 (3)	C12—H12A	0.9700
C2—H2	0.9300	C12—H12B	0.9700
C3—C4	1.403 (3)	O7—H7A	0.859 (9)
C3—C7	1.473 (3)	O7—H7B	0.855 (9)
C4—C5	1.389 (3)		
			
O3—S1—O1	114.30 (10)	O5—C7—C3	122.69 (19)
O3—S1—O2	111.89 (9)	O4—C7—C3	114.48 (18)
O1—S1—O2	111.36 (10)	N1—C8—C9	110.2 (2)
O3—S1—C1	107.72 (9)	N1—C8—H8A	109.6
O1—S1—C1	105.64 (9)	C9—C8—H8A	109.6
O2—S1—C1	105.25 (9)	N1—C8—H8B	109.6
C7—O4—H4	109.9 (19)	C9—C8—H8B	109.6
C4—O6—H6	105 (2)	H8A—C8—H8B	108.1
C8—N1—C12	112.9 (2)	C8—C9—C10	111.5 (2)
C8—N1—H1A	109.1 (15)	C8—C9—H9A	109.3
C12—N1—H1A	109.4 (16)	C10—C9—H9A	109.3
C8—N1—H1B	103.9 (16)	C8—C9—H9B	109.3
C12—N1—H1B	110.4 (16)	C10—C9—H9B	109.3
H1A—N1—H1B	111 (2)	H9A—C9—H9B	108.0
C2—C1—C6	119.45 (18)	C11—C10—C9	110.2 (2)
C2—C1—S1	120.57 (15)	C11—C10—H10A	109.6
C6—C1—S1	119.95 (15)	C9—C10—H10A	109.6
C1—C2—C3	120.67 (18)	C11—C10—H10B	109.6
C1—C2—H2	119.7	C9—C10—H10B	109.6
C3—C2—H2	119.7	H10A—C10—H10B	108.1
C2—C3—C4	119.16 (18)	C12—C11—C10	111.7 (2)
C2—C3—C7	121.05 (18)	C12—C11—H11A	109.3
C4—C3—C7	119.78 (18)	C10—C11—H11A	109.3
O6—C4—C5	117.99 (18)	C12—C11—H11B	109.3
O6—C4—C3	122.22 (19)	C10—C11—H11B	109.3
C5—C4—C3	119.79 (19)	H11A—C11—H11B	107.9
C6—C5—C4	120.09 (19)	N1—C12—C11	111.0 (2)
C6—C5—H5	120.0	N1—C12—H12A	109.4
C4—C5—H5	120.0	C11—C12—H12A	109.4
C5—C6—C1	120.8 (2)	N1—C12—H12B	109.4
C5—C6—H6A	119.6	C11—C12—H12B	109.4
C1—C6—H6A	119.6	H12A—C12—H12B	108.0
O5—C7—O4	122.83 (19)	H7A—O7—H7B	113.6 (15)
			
O3—S1—C1—C2	−17.95 (18)	C3—C4—C5—C6	1.0 (3)
O1—S1—C1—C2	−140.49 (16)	C4—C5—C6—C1	0.2 (3)
O2—S1—C1—C2	101.57 (16)	C2—C1—C6—C5	−1.1 (3)
O3—S1—C1—C6	164.09 (15)	S1—C1—C6—C5	176.88 (15)
O1—S1—C1—C6	41.55 (18)	C2—C3—C7—O5	−177.13 (18)
O2—S1—C1—C6	−76.39 (17)	C4—C3—C7—O5	3.7 (3)
C6—C1—C2—C3	0.9 (3)	C2—C3—C7—O4	3.2 (3)
S1—C1—C2—C3	−177.12 (14)	C4—C3—C7—O4	−175.97 (18)
C1—C2—C3—C4	0.3 (3)	C12—N1—C8—C9	55.3 (3)
C1—C2—C3—C7	−178.85 (17)	N1—C8—C9—C10	−55.7 (3)
C2—C3—C4—O6	178.52 (17)	C8—C9—C10—C11	56.0 (3)
C7—C3—C4—O6	−2.3 (3)	C9—C10—C11—C12	−55.3 (3)
C2—C3—C4—C5	−1.2 (3)	C8—N1—C12—C11	−54.9 (3)
C7—C3—C4—C5	177.93 (17)	C10—C11—C12—N1	54.5 (3)
O6—C4—C5—C6	−178.76 (18)		

### Hydrogen-bond geometry (Å, °)

**Table:**

*D*—H···*A*	*D*—H	H···*A*	*D*···*A*	*D*—H···*A*
O7—H7B···O1	0.855 (9)	1.933 (10)	2.786 (2)	176 (2)
O7—H7A···O2^i^	0.859 (9)	1.912 (10)	2.770 (2)	177 (2)
N1—H1B···O5	0.88 (3)	2.55 (2)	2.983 (3)	110.6 (18)
N1—H1B···O7^ii^	0.88 (3)	2.16 (3)	2.996 (3)	157 (2)
N1—H1A···O2^iii^	0.92 (3)	1.90 (3)	2.807 (3)	170 (2)
O6—H6···O5	0.84 (3)	1.82 (3)	2.597 (2)	153 (3)
O4—H4···O7^ii^	0.85 (3)	1.75 (3)	2.601 (2)	179 (3)

Symmetry codes: (i) *x*+1, *y*, *z*; (ii) *x*−1/2, −*y*+3/2, *z*+1/2; (iii) −*x*, −*y*+2, −*z*+1.
